# TRPC6 channel activation promotes neonatal glomerular mesangial cell apoptosis via calcineurin/NFAT and FasL/Fas signaling pathways

**DOI:** 10.1038/srep29041

**Published:** 2016-07-07

**Authors:** Hitesh Soni, Adebowale Adebiyi

**Affiliations:** 1 Department of Physiology University of Tennessee Health Science Center, Memphis TN, USA

## Abstract

Glomerular mesangial cell (GMC) proliferation and death are involved in the pathogenesis of glomerular disorders. The mechanisms that control GMC survival are poorly understood, but may include signal transduction pathways that are modulated by changes in intracellular Ca^2+^ ([Ca^2+^]_i_) concentration. In this study, we investigated whether activation of the canonical transient receptor potential (TRPC) 6 channels and successive [Ca^2+^]_i_ elevation alter neonatal GMC survival. Hyperforin (HF)-induced TRPC6 channel activation increased [Ca^2+^]_i_ concentration, inhibited proliferation, and triggered apoptotic cell death in primary neonatal pig GMCs. HF-induced neonatal GMC apoptosis was not associated with oxidative stress. However, HF-induced TRPC6 channel activation stimulated nuclear translocation of the nuclear factor of activated T-cells, cytoplasmic 1 (NFATc1). HF also increased cell death surface receptor Fas ligand (FasL) level and caspase-8 activity in the cells; effects mitigated by [Ca^2+^]_i_ chelator BAPTA, calcineurin/NFAT inhibitor VIVIT, and TRPC6 channel knockdown. Accordingly, HF-induced neonatal GMC apoptosis was attenuated by BAPTA, VIVIT, Fas blocking antibody, and a caspase-3/7 inhibitor. These findings suggest that TRPC6 channel-dependent [Ca^2+^]_i_ elevation and the ensuing induction of the calcineurin/NFAT, FasL/Fas, and caspase signaling cascades promote neonatal pig GMC apoptosis.

Glomerular mesangial cell (GMC) proliferation and death are involved in the maintenance of glomerular integrity and pathophysiological mechanisms that underlie kidney dysfunctions^1^. GMC apoptosis contributes to the resolution of glomerular hypercellularity, a common characteristic of proliferative glomerulonephritis^2^. Extracellular matrix accumulation, GMC apoptosis, and glomerular sclerosis are associated with proteinuria and hypertension in diabetic nephropathy^1^,^3^,^4^. Mesangial integrity is also altered in childhood nephrotic syndrome^5^. The mechanisms that regulate GMC survival are unresolved, but may include signal transduction pathways that are modulated by changes in intracellular Ca^2+^ ([Ca^2+^]_i_) concentration^6^.

Ion channels, including voltage-dependent Ca^2+^, Ca^2+^-activated K^+^, Ca^2+^-activated Cl^−^, and transient receptor potential channels are functionally expressed in GMCs^7^,^8^. These channels control [Ca^2+^]_i_ concentration and hence, Ca^2+^-sensitive cellular events, including contraction, proliferation, and apoptosis^7^,^8^. The canonical transient receptor potential (TRPC) 6 has been implicated in glomerular pathophysiology^9^,^10^. TRPC6 channel activation alters podocyte survival and actin cytoskeleton dynamics^9^,^10^. Focal segmental glomerulosclerosis, an important cause of nephrotic syndrome is associated with TRPC6 channel gain of function mutations and succeeding elevation in TRPC6-dependent Ca^2+^ influx in podocytes^11^,^12^. An increase in Ca^2+^ influx elicited by angiotensin II-induced TRPC6 channel activation in podocytes has also been reported in diabetic nephropathy^13^. By contrast, TRPC6 channel expression and angiotensin II-induced [Ca^2+^]_i_ elevation are downregulated in high glucose-challenged GMCs^14^. Studies have also shown that TRPC6-mediated [Ca^2+^]_i_ elevation regulates angiotensin II- and phenylephrine-induced proliferation and chronic hypoxia-induced actin assembly and reorganization in GMCs^15^,^16^,^17^. However, the downstream targets that link TRPC6-dependent Ca^2+^ signaling to cellular events in GMCs are poorly understood.

The nuclear factor of activated T cells (NFAT) family of transcription factors includes four members whose activations are regulated by calcineurin, a Ca^2+^-dependent protein phosphatase^18^,^19^,^20^. NFATs control transcription of a variety of genes, including those involved in cell differentiation, growth, and death^18^,^19^,^20^. In cardiac cells and podocytes, NFATs are targets of TRPC6-dependent [Ca^2+^]_i_ elevation^21^,^22^,^23^,^24^. However, whether NFATs are downstream effectors of TRPC6 channel activation in GMCs is unclear. Given that NFAT-regulated genes control cell survival^18^,^19^,^20^, we examined whether a direct activation of TRPC6 channels alters neonatal GMC survival via NFAT signaling pathway. Our data suggest that hyperforin (HF)-induced TRPC6 activation inhibits proliferation and promotes apoptosis of primary neonatal pig GMCs. We also show that TRPC6-mediated neonatal GMC apoptosis is associated with an induction of the cell death surface receptor Fas ligand (FasL) and caspase-8 by NFATc1. Collectively, we provide a novel insight into the mechanisms by which TRPC6 channel-dependent [Ca^2+^]_i_ elevation and sequential activation of the calcineurin/NFAT and FasL/Fas signaling pathways stimulate neonatal pig GMC apoptosis.

## Results

### HF-induced TRPC6 channel activation elevates [Ca^2+^]_i_ in neonatal GMCs

**Figure 1:**
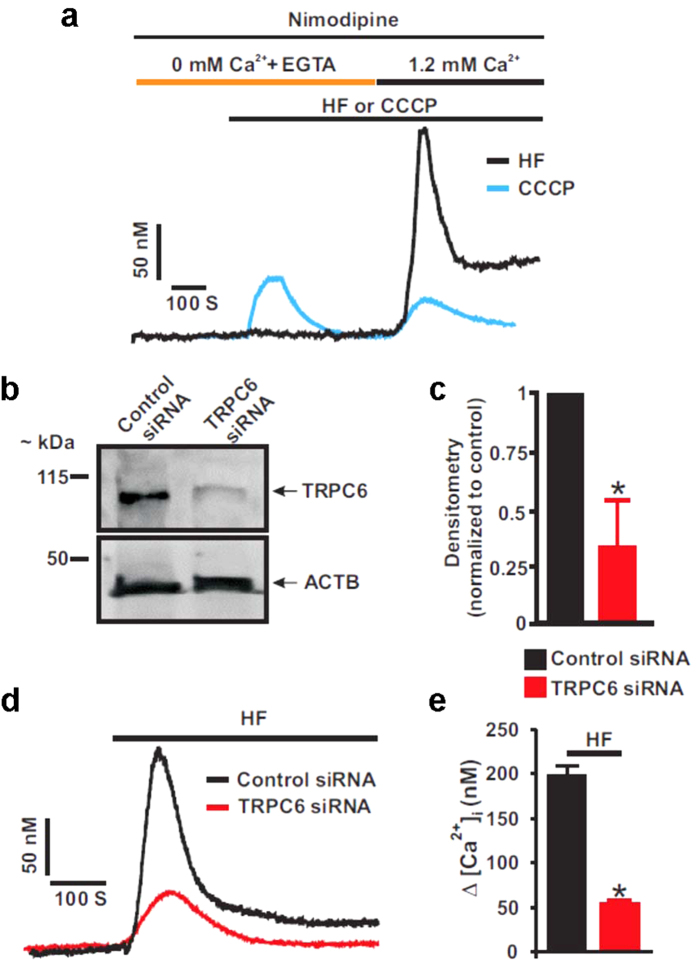
HF-induced TRPC6 channel activation elevates [Ca^2+^]_i_ in neonatal GMCs. (**a**) Exemplar traces showing the effects of HF and CCCP on [Ca^2+^]_i_ concentration in the absence and presence of extracellular Ca^2+^ in neonatal pig GMCs. The bathing solution was supplemented with EGTA (0.1 mM) and nimodipine (1 μM) to chelate Ca^2+^ and block L-type Ca^2+^ channels, respectively. (**b,c**) Western blot image and mean data (n = 3 each) confirming siRNA-mediated knockdown of TRPC6 channels. (**d,e**) Traces and mean data indicating that TRPC6 channel knockdown attenuates HF (10 μM)-induced [Ca^2+^]_i_ elevation in neonatal GMCs (n = 4 each). *P < 0.05 vs. control siRNA.

TRPC6 channels regulate [Ca^2+^]_i_ concentration in rat and human GMCs^14^,^15^,^16^,^17^. To confirm that activation of TRPC6 channels stimulates Ca^2+^ influx in neonatal pig GMCs, we studied the effect of HF, a TRPC6 channel activator^25^,^26^,^27^,^28^,^29^,^30^ on [Ca^2+^]_i_ concentration in the cells. First, we examined whether HF stimulates Ca^2+^ release from intracellular Ca^2+^ stores in the cells. In the absence of extracellular Ca^2+^, HF did not alter basal [Ca^2+^]_i_ in the cells (Fig. 1a). However, successive re-addition of extracellular Ca^2+^ in the continued presence of HF resulted in an increase in [Ca^2+^]_i_ by 186.7 ± 3.4 nM (n = 3; Fig. 1a). By contrast, in the absence of extracellular Ca^2+^, protonophore carbonyl cyanide m-chlorophenyl hydrazine (CCCP) elevated [Ca^2+^]_i_ by 64.4 ± 9.2 nM (n = 5; Fig. 1a) in the cells. Restoration of extracellular Ca^2+^ in the presence of CCCP did not stimulate a further increase in [Ca^2+^]_i_ (29.2 ± 6.0 nM; n = 5; P < 0.05 vs. absence of extracellular Ca^2+^; Fig. 1a). Next, we knocked down TRPC6 channels and examined the effect of HF on [Ca^2+^]_i_ concentration in the cells. As shown in Fig. 1b,c, TRPC6 siRNA reduced TRPC6 protein expression by ~66%. HF increased [Ca^2+^]_i_ by ~197 nM in control cells (Fig. 1d,e). However, TRPC6 channel knockdown significantly reduced HF-induced [Ca^2+^]_i_ elevation (Fig. 1d,e). These data indicate that HF does not stimulate Ca^2+^ release from intracellular organelles, but elevates [Ca^2+^]_i_ by activating membrane-resident TRPC6 channels in neonatal pig GMCs.

### TRPC6 channel activation inhibits proliferation and promotes apoptosis of neonatal GMC

**Figure 2:**
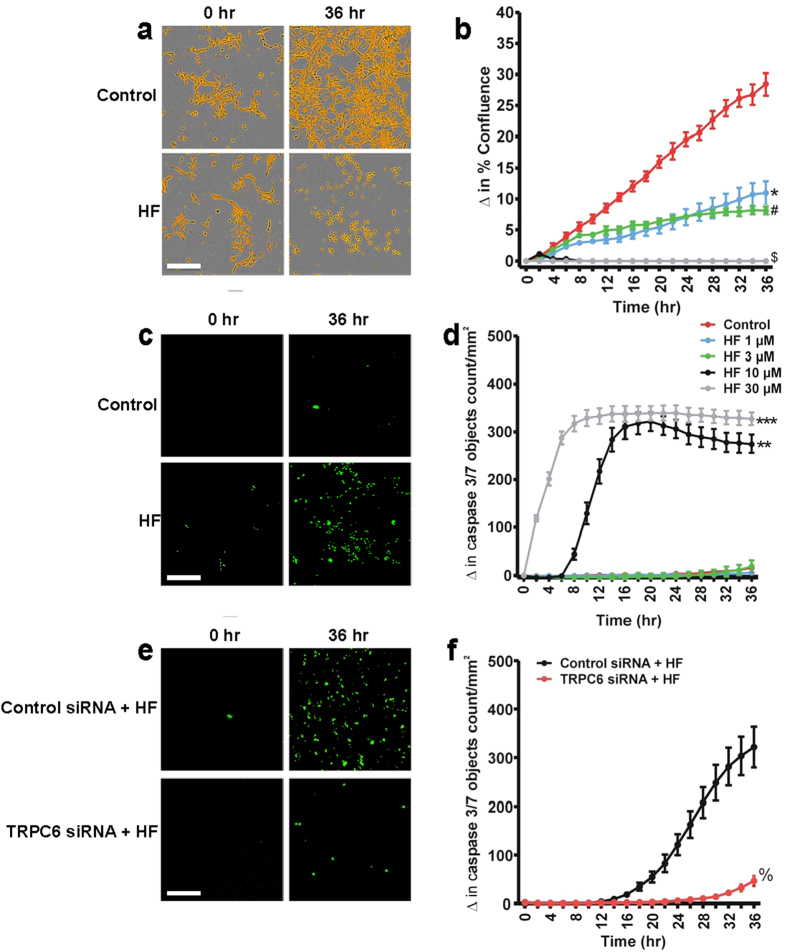
TRPC6 channel activation inhibits proliferation and promotes apoptosis of neonatal GMCs. (**a**) Phase contrast images (segmentation mask illustrated in goldenrod; HF, 10 μM) and (**b**) Cell growth curves showing time- and concentration-dependent anti-proliferative effect of HF (n = 4 each) in neonatal pig GMCs. (**c**) Images (green fluorescent staining of nuclear DNA in apoptotic cells; HF, 10 μM) and (**d**) Kinetic curves demonstrating that HF (n = 4 each) induces time- and concentration-dependent increase in caspase-3/7 activity in neonatal pig GMCs. (**e**) Images and (**f**) Kinetic curves illustrating that TRPC6 channel knockdown attenuates HF (10 μM; n = 4 each)-induced caspase-3/7 activity in neonatal pig GMCs. ^#^P < 0.05 vs. control (DMSO; 12–36 hr); *P < 0.05 vs. control (DMSO; 10–36 hr); ^$^P < 0.05 vs. control (DMSO; 6–36 hr); **P < 0.05 vs. control (DMSO; 10–36 hr); ***P < 0.05 vs. control (DMSO; 2–36 hr); ^%^P < 0.05 vs. control siRNA (20–36 hr); Scale bars = 100 μm.

Changes in [Ca^2+^]_i_ regulate several intracellular signaling pathways that promote GMC proliferation and death^6^,^31^. Data here indicate that HF stimulates [Ca^2+^]_i_ elevation via TRPC6 channels (Fig. 1). To study the effect of HF on GMC survival, we measured the time-course of proliferation and apoptotic death in the cells. The CellPlayer caspase-3/7 green fluorescence reagent is non-fluorescent in non-apoptotic cells. However, activation of caspase activity during apoptosis increases the fluorescent signal. As shown in Fig. 2a,b, HF inhibited neonatal pig GMC confluence in a time- and concentration-dependent manner. At the same time, HF increased caspase-3/7 activity in the cells (Fig. 2c,d). At 1 and 3 μM, HF inhibited GMC proliferation but did not significantly induce apoptosis (Fig. 2b,d). To confirm that TRPC6 channels mediate HF-induced neonatal pig GMC apoptosis, we measured caspase-3/7 activity in cells transfected with a non-targeting control and TRPC6 channel siRNAs. As shown in Fig. 2e,f, knockdown of TRPC6 channels attenuated HF-induced caspase-3/7 activation in the cells. Together, these data suggest that HF-induced TRPC6 channel activation inhibits proliferation and stimulates apoptosis of neonatal pig GMCs.

### HF-induced neonatal GMC apoptosis is not associated with oxidative stress

**Figure 3:**
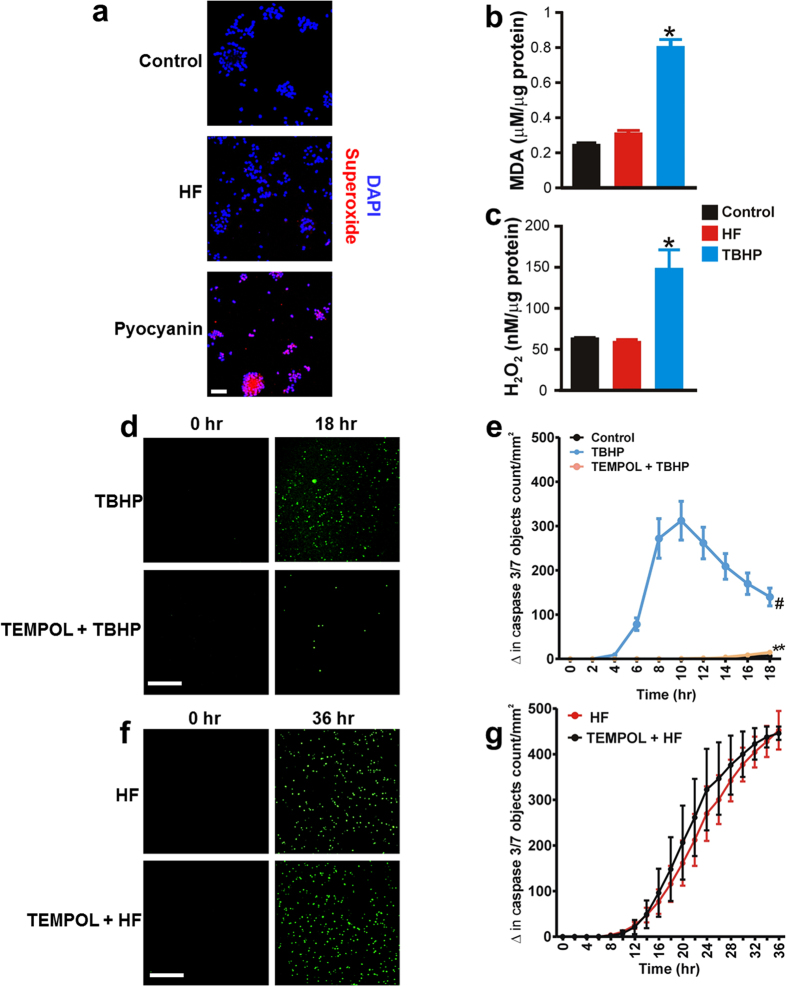
HF-induced neonatal GMC apoptosis is not associated with oxidative stress. (**a**) Representative confocal images showing that superoxide production was detected in GMCs treated with ROS inducer pyocyanin (10 μM; 8 hr)-, but not in HF (10 μM; 8 hr)-treated cells (images were obtained from 3 coverslips per experimental condition). (**b**) Mean data illustrating that unlike tert-butyl hydroperoxide (TBHP; 10 μM; 8 hr), HF (10 μM; 8 hr) does not increase MDA production in neonatal pig GMCs (n = 4 each). (**c**) Mean data showing cellular H_2_O_2_ concentration in DMSO (control)-, HF (10 μM; 8 hr)- and TBHP (10 μM; 8 hr)-treated cells (n = 5 each). (**d**) Images (green fluorescent staining of nuclear DNA in apoptotic cells) and (**e**) Kinetic curves indicating that TBHP (10 μM; n = 5 each)-induced caspase-3/7 activity in neonatal pig GMCs is inhibited by TEMPOL. (**f**) Images and (**g**) Kinetic curves demonstrating that HF (10 μM; n = 4 each)-induced caspase-3/7 activity in neonatal pig GMCs is unaltered by TEMPOL. *P < 0.05 vs. DMSO and HF; ^#^P < 0.05 vs. Control (PBS; 6–18 hr); **P < 0.05 vs. TBHP (6–18 hr); Scale bars = 100 μm.

To investigate the role of oxidative stress in HF-induced neonatal GMC apoptosis, we first measured reactive oxygen species (ROS) generation in control and neonatal pig GMCs treated with HF. Confocal microscopy showed that pyocyanin, a superoxide inducer stimulates superoxide generation in neonatal pig GMCs (Fig. 3a). By contrast, neither DMSO (control) nor HF caused significant superoxide generation in the cells (Fig. 3a). Thiobarbituric acid reactive substance (TBARS) assay indicated that unlike oxidant tert-butyl hydroperoxide (TBHP), HF did not increase malondialdehyde (MDA; an end product of lipid peroxidation) in the cells (Fig. 3b). Whereas HF did not alter H_2_O_2_ level, TBHP elevated H_2_O_2_ production in the cells (Fig. 3c). Next, we examined whether inhibition of oxidative stress mitigates HF-induced neonatal pig GMC apoptosis. As shown in Fig. 3d,e, TBHP promoted rapid apoptosis of the cells. TEMPOL, a cell permeable ROS scavenger essentially abolished TBHP-induced GMC apoptosis (Fig. 3d,e). By contrast, TEMPOL did not alter HF-induced apoptosis of the cells (Fig. 3f,g). These findings indicate that HF-induced neonatal pig GMC apoptosis is not associated with oxidative stress.

### TRPC6 channel-dependent [Ca^2+^]_i_ elevation stimulates nuclear translocation of NFATc1 in neonatal GMCs

**Figure 4:**
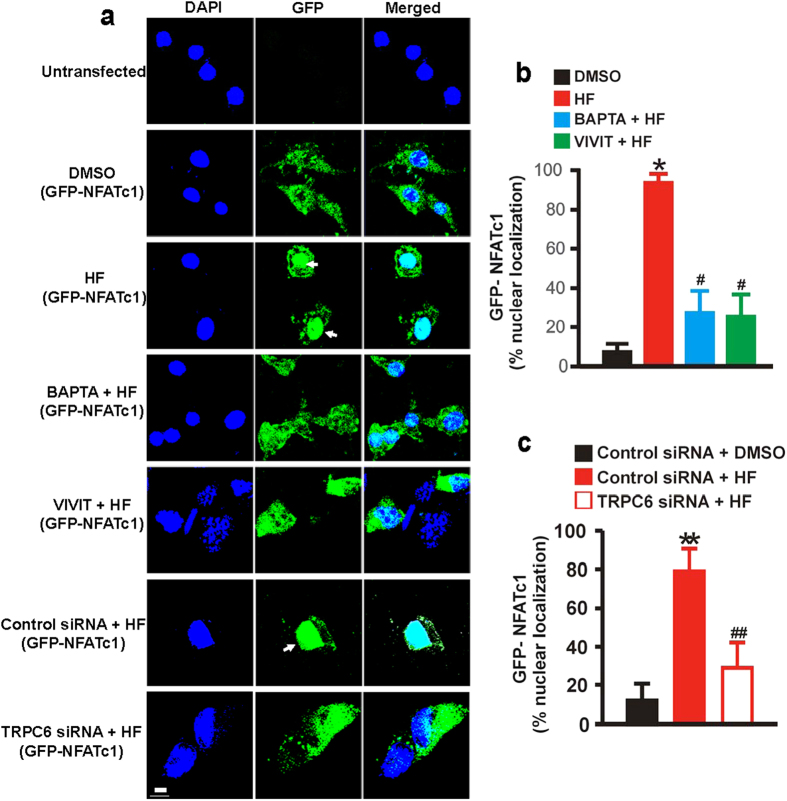
TRPC6 channel-dependent [Ca^2+^]_i_ elevation stimulates nuclear translocation of NFATc1 in neonatal GMCs. (**a**) Confocal microscopy images, and (**b,c**) Mean data showing that HF (10 μM; 1 hr) stimulates nuclear translocation of GFP-tagged NFATc1 in neonatal pig GMCs and that BAPTA (1 μM), VIVIT (1 μM), and TRPC6 channel knockdown inhibit HF-induced NFATc1 nuclear translocation (n = 9, DMSO, HF, BAPTA + HF, and VIVIT + HF; n = 10, control siRNA + DMSO; n = 12 control siRNA + HF and TRPC6 siRNA + HF image fields from 3 coverslips per experimental condition). Cells were pretreated with blockers/inhibitors for ~30 min before the addition of HF. *P < 0.05 vs. DMSO; ^#^P < 0.05 vs. HF; **P < 0.05 vs. control siRNA + DMSO; ^##^P < 0.05 vs. control siRNA + HF; Sacle bar = 20 μm. White arrows indicate NFATc1 nuclear localization.

To examine whether HF-induced [Ca^2+^]_i_ elevation via TRPC6 channels stimulates nuclear translocation of NFATc1 in neonatal GMCs, we studied the localization of a GFP-NFATc1 cDNA clone that was transiently transfected into neonatal pig GMCs. Unlike DMSO (control)-treated cells, GFP-NFATc1 showed predominant nuclear localization in HF-treated cells (Fig. 4a,b). GFP-NFATc1 nuclear localization in the cells was significantly reduced by BAPTA, an [Ca^2+^]_i_ chelator and VIVIT, a calcineurin/NFAT inhibitor (Fig. 4a,b). Also, GFP-NFATc1 nuclear localization was attenuated by TRPC6 channel knockdown (Fig. 4a,c). These data suggest that HF-induced [Ca^2+^]_i_ elevation via TRPC6 channels elicits nuclear translocation of NFATc1 in neonatal pig GMCs.

### TRPC6 channel-mediated [Ca^2+^]_i_ elevation increases FasL level and caspase-8 activity in neonatal GMCs

**Figure 5:**
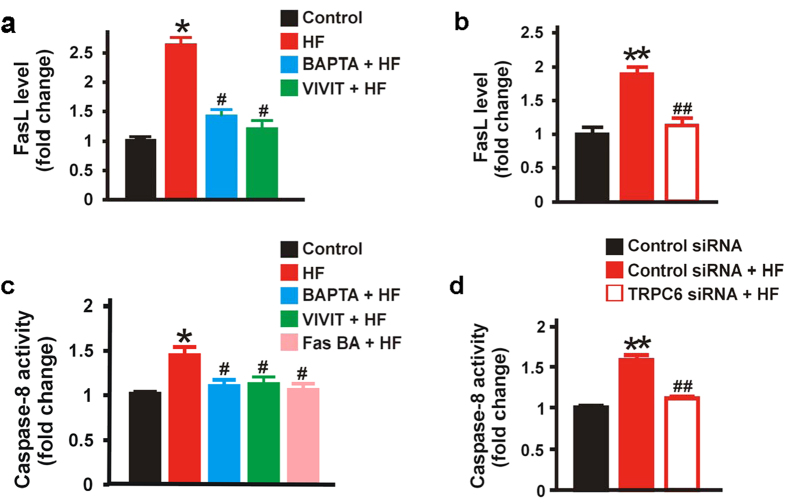
TRPC6 channel-mediated [Ca^2+^]_i_ elevation increases FasL level and caspase-8 activity in neonatal GMCs. (**a**) Mean data obtained from porcine FasL ELISA assay demonstrating that HF (10 μM; 8 hr) elevates FasL level in neonatal pig GMCs and that HF-induced FasL is abrogated by BAPTA (1 μM) and VIVIT (1 μM); n = 6 each. (**b**) Mean data showing that HF (10 μM; 8 hr)-induced FasL elevation in neonatal pig GMCs is reduced by siRNA-mediated TRPC6 channel knockdown; n = 6 each. (**c**) Mean data obtained from caspase-8 colorimetric assay indicating that HF (10 μM; 8 hr) increases caspase-8 activity in neonatal GMCs, and that BAPTA (1 μM), VIVIT (1 μM), and Fas blocking antibody (Fas BA; 10 μg/mL; n = 4 each) essentially abolish HF-induced increase in caspase-8 activity. (**d**) Mean data showing that HF (10 μM; 8 hr)-induced caspase-8 activity in neonatal pig GMCs is attenuated by siRNA-mediated TRPC6 channel knockdown (n = 4, control siRNA and control siRNA + HF; n = 5, TRPC6 siRNA + HF). Cells were pretreated with blockers/inhibitors for ~30 min before the addition of HF. Data are expressed as fold change relative to the control. *P < 0.05 vs. DMSO; ^#^P < 0.05 vs. HF; **P < 0.05 vs. control siRNA; ^##^P < 0.05 vs. control siRNA + HF.

FasL/Fas interaction at the cell surface is a major contributor to death-inducing signaling pathways in many cell types, including GMCs^32^,^33^. FasL/Fas interaction triggers caspase-8 and -3 activation^32^. Our data indicate that HF-induced TRPC6 activation stimulates NFATc1 nuclear localization in neonatal pig GMCs (Fig. 4). Given that NFATs are involved in transcriptional regulation of FasL expression^34^,^35^, we reasoned that TRPC6-depedent NFATc1 activation may elevate membrane FasL level and stimulate caspase-8 activity in neonatal GMCs. Hence, we used a porcine FasL ELISA kit to quantify the level of FasL in neonatal pig GMC lysates. We also measured caspase-8 activity in the cell lysates. As shown in Fig. 5a, FasL level was increased in neonatal pig GMCs treated with HF. HF-induced increase in FasL was diminished in cells pretreated with BAPTA and VIVIT (Fig. 5a). siRNA-mediated knockdown of TRPC6 channels also reduced HF-induced increase in FasL level (Fig. 5b). Similarly, HF treatment elevated caspase-8 activity in neonatal pig GMCs (Fig. 5c). HF-induced elevation in caspase-8 activity was significantly attenuated by BAPTA, VIVIT, and TRPC6 channel knockdown (Fig. 5c,d). To examine the role of FasL/Fas activation in HF-induced caspase-8 activity, we blocked FasL/Fas interaction with a Fas blocking antibody (Fas BA). As shown in Fig. 5c, there was no significant change in caspase-8 activity in GMCs, pretreated with Fas BA before HF application. These findings suggest that TRPC6-dependent [Ca^2+^]_i_ elevation in neonatal pig GMCs increases FasL and caspase-8 activity via calcineurin/NFAT signaling. Our data also demonstrate that FasL/Fas interaction regulates HF-induced caspase-8 activation in the cells.

### TRPC6 channel-dependent [Ca^2+^]_i_ elevation and successive activation of the NFAT and FasL/Fas signaling pathways trigger neonatal GMC apoptosis

**Figure 6:**
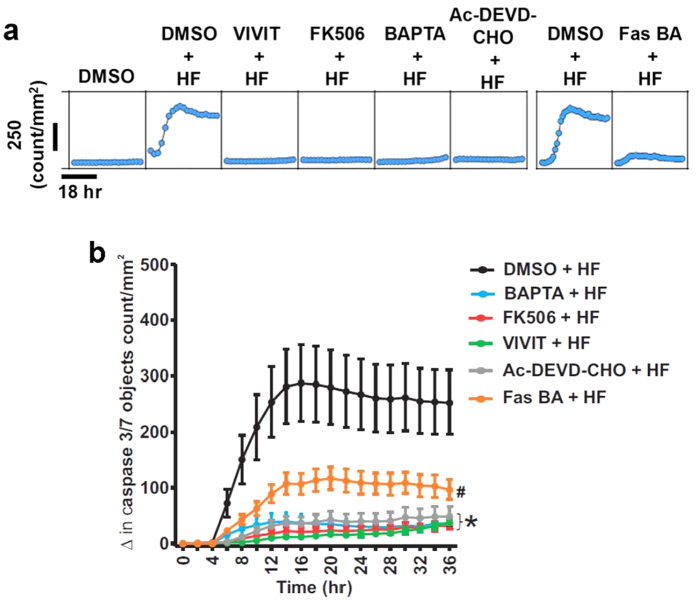
TRPC6 channel-dependent [Ca^2+^]_i_ elevation and successive activation of the NFAT and FasL/Fas signaling pathways trigger neonatal GMC apoptosis. (**a**) Representative microplate graphs and (**b**) Kinetic curves showing HF-induced caspase-3/7 activity in control and BAPTA (1 μM; n = 10)-, FK506 (100 nM; n = 10)-, VIVIT (1 μM; n = 10)-, Ac-DEVD-CHO (50 μM; n = 10)-, and Fas BA (10 μg/mL; n = 5)-treated GMCs. Cells were pretreated with blockers/inhibitors for ~30 min before the addition of HF. *P < 0.05 vs. control (8–36 hr); ^#^P < 0.05 vs. control (10–36 hr).

**Figure 7:**
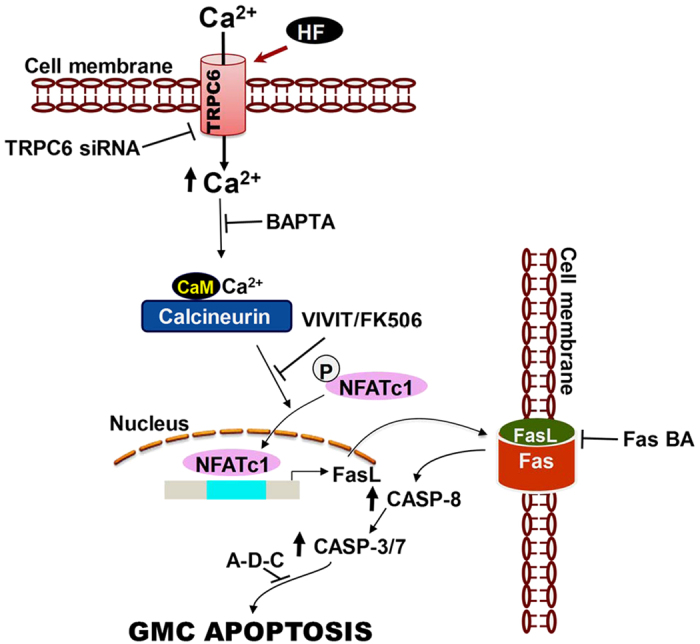
Schematic diagram illustrating hypothetical mechanisms by which an elevation in [Ca^2+^]_i_ as a result of TRPC6 channel activation, and subsequent induction of the calcineurin/NFAT, FasL/Fas, and caspase signaling cascades promote neonatal GMC apoptosis. CASP (caspase); Ac-DEVD-CHO (A-D-C); BA (blocking antibody); CaM (calmodulin).

To confirm that TRPC6 channel-dependent [Ca^2+^]_i_ elevation and successive activation of the extrinsic apoptotic signaling pathway induce neonatal GMC apoptosis, we measured HF-induced caspase-3/7 activation in neonatal pig GMCs pretreated with DMSO (control), BAPTA, and inhibitors of NFAT, Fas, and caspase-3/7. As shown in Fig. 6, HF-induced caspase-3/7 activation was mitigated by BAPTA, VIVIT, FK506 (a calcineurin inhibitor), Fas BA, and Ac-DEVD-CHO (a caspase-3/7 inhibitor). Together, these data demonstrate that an elevation in [Ca^2+^]_i_ elicited by HF-induced TRPC6 channel activation, and sequential stimulation of the calcineurin/NFAT, FasL/Fas, and caspase signaling pathways promote neonatal GMC apoptosis (Fig. 7).

## Discussion

The fine-tuning of the expression and function of ion channels and second messengers that maintain [Ca^2+^]_i_ concentration underlies a variety of signaling events that control cell survival^6^. HF, a phloroglucinol derivative and selective TRPC6 channel activator elevated [Ca^2+^]_i_ in neuronal, vascular smooth muscle, and dermal cells and cardiac fibroblast^25^,^26^,^27^,^28^. HF impedes tumor cell growth by inducing caspase-dependent apoptosis^36^,^37^. However, modulation of GMC survival by HF-induced TRPC6 activation has not been previously investigated. In this study, we show for the first time that activation of TRPC6 channels by HF increases [Ca^2+^]_i_ concentration and induces NFAT-, FasL/Fas-, and caspase-dependent neonatal pig GMC apoptosis.

The control of mesangial [Ca^2+^]_i_ homeostasis involves TRPC channel-mediated signal transduction pathways^8^,^38^. TRPC channels, comprising of seven members (TRPC1-7), can assemble as homo- or hetero-tetramers to form non-selective cation channels that are activated by multiple signaling modalities, including membrane stretch, G-protein-coupled receptor stimulation, and [Ca^2+^]_i_ store depletion^39^,^40^. Previous studies have shown that TRPC6 channels participate in GMC proliferation associated with angiotensin II- and phenylephrine-induced store- and receptor-operated Ca^2+^ entry in rat and human GMC lines^15^,^16^. Here, simultaneous measurement of cell confluence metrics and caspase-3/7 activity in real-time indicated that a direct activation of TRPC6 channels inhibits proliferation and triggers apoptosis of primary neonatal pig GMCs. The conflicting data on the effect of TRPC6 activation on GMC growth may be related to kidney maturation or the different mechanisms of channel activation. Whether [Ca^2+^]_i_ elevation induces GMC proliferation or death may also depend on downstream cellular signaling pathways that are activated^6^.

To investigate the mechanisms that mediate HF-induced neonatal GMC apoptosis, we tested the hypothesis that HF promotes oxidative stress in the cells. Our data show that at the concentration that elicited apoptotic cell death, HF did not stimulate lipid peroxidation and superoxide and H_2_O_2_ production in the cells. Furthermore, at the concentration that abrogated TBHP-induced apoptosis, ROS scavenger TEMPOL did not alter HF-induced apoptosis of the cells. Hence, oxidative damage is an unlikely mechanism of HF-induced neonatal pig GMC apoptosis.

The NFAT family of transcription factors are important regulators of genes that promote differentiation, proliferation and death in a broad range of cells^18^,^19^,^20^. In resting cells, phosphorylated NFATs are mostly localized in the cytoplasm, and are activated by calcineurin, a Ca^2+^/calmodulin (CaM)-dependent serine/threonine phosphatase^18^,^19^,^20^. Following an elevation in [Ca^2+^]_i_, the Ca^2+^-CaM complex activates calcineurin. NFAT proteins are then directly dephosphorylated by activated calcineurin, resulting in their nuclear translocation and induction of NFAT-mediated gene transcription^18^,^19^,^20^. In cardiac cells, NFAT stimulation and the resultant pathological hypertrophy are associated with [Ca^2+^]_i_ increase caused by the activation of TRPC1, TRPC3, TRPC4, and TRPC6 channels^22^,^41^,^42^. In the kidney, TRPC6 channel-mediated activation of NFATc1-dependent gene transcription is involved in glomerulosclerosis^23^,^24^. Tumor necrosis factor- and angiotensin II-induced activation of NFATc1 can also increase TRPC6 channel expression in podocytes^21^,^43^. Here, we show for the first time that activation of TRPC6 channels in neonatal GMCs stimulates NFATc1 nuclear translocation in a Ca^2+^-dependent fashion. HF-induced apoptotic cell death was also reduced in neonatal GMCs pretreated with calcineurin/NFAT inhibitors FK506 and VIVIT. These findings indicate that NFAT is a downstream target of TRPC6 channel-dependent [Ca^2+^]_i_ elevation in GMCs. The NFAT family consists of four Ca^2+^/calcineurin-responsive members NFATc1, NFATc2, NFATc3, and NFATc4^18^,^19^,^20^. Thus, activation of multiple NFAT members by TRPC6-mediated [Ca^2+^]_i_ elevation in GMCs is possible, but not demonstrated in this study.

Activation of the cell death surface receptor, Fas by its cognate ligand FasL is a major contributor to the extrinsic pathway of programmed cell death^32^,^34^,^44^. FasL/Fas interaction recruits the adapter protein Fas-associated protein with death domain, which in turn employs its death effector domain to induce procaspase-8 and -10^45^,^46^,^47^. Formation of the death-inducing signaling complex as a result of clustering and subsequent autocatalysis of procaspase-8 and -10 triggers apoptosis via downstream executioner caspases, including caspase-3 and -7^45^,^47^,^48^. The expression of FasL is controlled by transcription factors such as nuclear factor-kappa B, early growth response protein, and NFAT^34^,^35^. NFAT-induced FasL expression has been implicated in Jurkat T, breast cancer, cardiac, neuronal, and Leydig cell apoptosis^49^,^50^,^51^,^52^, but whether NFAT regulates cell death induced by the FasL/Fas pathway in GMCs was unclear. Data here show that TRPC6 activation elevates membrane FasL level and caspase-8 activity in neonatal pig GMCs, and these effects were essentially abolished by [Ca^2+^]_i_ chelation, calcineurin/NFAT inhibition, and TRPC6 channel knockdown. These data suggest the involvement of TRPC6-dependent calcineurin/NFAT activation in FasL and caspase-8 induction in neonatal pig GMCs. HF-induced caspase-3/7 activity was also attenuated by [Ca^2+^]_i_ chelation, calcineurin/NFAT inhibition, and Fas blockade. Together, these findings indicate that TRPC6 channel-mediated [Ca^2+^]_i_ elevation and subsequent activation of calcineurin/NFAT, FasL/Fas, and caspase cascades result in neonatal pig GMC apoptosis.

Studies have suggested that HF exhibit protonophore activity, and may stimulate Ca^2+^ release from the mitochondria, independently of TRPC6 channels^53^,^54^,^55^. Protonophores, including CCCP, inhibit mitochondrial Ca^2+^ uptake and induce mitochondrial Ca^2+^ release, thereby increasing [Ca^2+^]_i_^56^,^57^. Here, we show that in neonatal pig GMCs, CCCP elevated [Ca^2+^]_i_ which was independent of extracellular Ca^2+^, suggesting Ca^2+^ release from the intracellular stores. By contrast, HF did not stimulate Ca^2+^ release from the intracellular stores but induced nimodipine-insensitive and TRPC6-dependent Ca^2+^ influx in the cells. HF-induced NFATc1 nuclear translocation, FasL and caspase 8 activation, and apoptosis of neonatal pig GMCs were also dependent on TRPC6 channels. These data suggest that TRPC6 channels mediate the effects of HF reported in the present study. Perhaps, the molecular mechanisms underlying the pharmacological actions of HF is dependent on cell type. Also, data in this study were derived from primary neonatal GMCs. Thus, TRPC6 channel-mediated intracellular signaling may differ in adult GMCs.

Whereas the contribution of TRPC6 channels to podocyte pathology is well known, their role in mesangial pathophysiology is poorly understood. Given the involvement of GMC apoptosis in glomerulopathy, we propose that mesangial derangement promoted by TRPC6-dependent [Ca^2+^]_i_ elevation and subsequent cell death may be of importance in renal dysfunctions, especially in immature kidneys.

## Methods

### Primary GMC culture

Animal welfare and use in this study were in compliance with the National Institutes of Health Guide for the Care and Use of Laboratory Animals. All experimental protocols were approved by and carried out in accordance with the guidelines and regulations of the Institutional Animal Care and Use Committee of the University of Tennessee Health Science Center (UTHSC). Male newborn pigs (1.5–2 kg) were purchased from Nichols Hog Farm (Olive Branch, MS) and maintained at the UTHSC Comparative Medicine Department. Renal glomeruli were isolated from the pigs (<1-week-old) by serial sieving of renal cortical homogenates using sterile stainless steel meshes. GMCs were then cultured from decapsulated glomeruli as we have previously described^58^.

### Western immunoblotting

Cultured GMCs were homogenized in ice-cold RIPA buffer supplemented with a protease inhibitor cocktail (Thermo Scientific, Rockford, IL). Following isolation, proteins were separated and detected as we have previously described^58^,^59^.

### siRNA and cDNA transfection

GMC suspensions were electroporated in single cuvettes containing pre-warmed culture medium and a pool of 2 target-specific TRPC6 siRNAs or a non-targeting control siRNA (Sigma-Aldrich, St. Louis, MO). An electrical field to induce siRNA transfection was applied using the ECM 830 square wave electroporation system (Harvard Apparatus, Holliston, MA). Ten minutes after electroporation, the cells were plated for ~72 hours. Western immunoblotting was used to confirm efficient knockdown. GFP-tagged human nuclear factor of activated T-cells, cytoplasmic 1 (NFATc1; OriGene Technologies, Rockville, MD) cDNA clone (400 ng/mL) was transfected into GMCs using the TurboFect transfection reagent (Life Technologies, Grand Island, NY). Transfected cells were maintained in 5% CO_2_ humidified incubator for 48 h at 37 °C. Images were acquired using a Zeiss laser-scanning confocal microscope.

### [Ca^2+^]_i_ imaging

Changes in [Ca^2+^]_i_ concentrations were measured in GMCs loaded with fura-2-acetoxymethyl ester using a ratiometric fluorescence system (Ionoptix Corp., Milton, MA, USA) as we have previously described^58^,^59^. To examine whether HF and CCCP induce Ca^2+^ release from intracellular stores, cells were incubated in Ca^2+^ free modified Krebs’ solution^58^,^59^ supplemented with 0.1 mM EGTA (a calcium chelator) and L-type Ca^2+^ channel blocker nimodipine (1 μM).

### Live content microscopy of GMCs

Real-time growth and kinetic quantification of apoptosis in GMCs seeded in microplates (5 × 10^3^ cells/well) were performed using CellPlayer caspase-3/7 reagent (Essen BioScience, Ann Arbor, MI) and the IncuCyte ZOOM live content microscopy system (Essen BioScience). Cell growth and kinetic activation of caspase-3/7 were monitored in real-time and automatically acquired at two-hourly intervals by the IncuCyte interface and software.

### Reactive oxygen species (ROS) determination

Lipid peroxidation was evaluated in cell lysates using the TBARS kit (Thermo Scientific, Rockford, IL, Cat. No. KA1381). Superoxide generation was examined using a superoxide fluorogenic kit that specifically detects superoxide levels in live cells (Enzo Life Sciences, Farmingdale, NY; Cat. No. ENZ-51012). The fluorescence generated by this probe was visualized and documented using a Zeiss laser-scanning confocal microscope. H_2_O_2_ concentration in the cells was measured using a colorimetric detection kit (Arbor Assay, Ann Arbor, MI, Cat. No. K034-H1).

### FasL and caspase-8 assays

GMCs were cultured in 6-well microplates as described above. Membrane FasL concentration and caspase-8 activity were quantified in cell lysates using the porcine FasL ELISA (NeoScientific, Woburn, MA, Cat. No. PF0058) and FLICE/caspase-8 colorimetric assay (BioVision, Inc. Milpitas, CA, Cat. No. K113-25) kits. Data obtained from the cell lysates were normalized to protein concentrations.

### Antibodies and chemicals

Mouse monoclonal anti-actin (Cat. No. ab3280) and rabbit polyclonal anti-TRPC6 (Cat. No. ab81111) primary antibodies and HRP-conjugated anti-rabbit and anti-mouse secondary antibodies were purchased from Abcam (Cambridge, MA). DMSO, hyperforin, and tert-Butyl hydroperoxide were purchased from Sigma-Aldrich. BAPTA, FK506, VIVIT, Ac-DEVD-CHO, Fas blocking antibody, ionomycin, pluronic F-127, and Fura-2 AM were purchased from Assay Biotechnology (Sunnyvale, CA), Cell Signaling Technology (Danvers, MA), Cayman Chemical Company (Ann Arbor, MI), Biotium Inc. (Hayward, CA), ProSpec-Tany TechnoGene Ltd (Rehovot, Israel), Cayman Chemical Company, Anaspec (Fremont, CA), and Life Technologies, respectively.

### Data analysis

All data are expressed as mean ± standard error of mean (SEM). Statistical significance was determined using Student’s t-tests for paired or unpaired data and analysis of variance with Bonferroni post hoc test for multiple comparisons. A P value < 0.05 was considered significant.

## Additional Information

**How to cite this article**: Soni, H. and Adebiyi, A. TRPC6 channel activation promotes neonatal glomerular mesangial cell apoptosis via calcineurin/NFAT and FasL/Fas signaling pathways. *Sci. Rep.* **6**, 29041; doi: 10.1038/srep29041 (2016).

## References

[b1] Schlondorff D. & Banas B. The mesangial cell revisited: no cell is an island. J. Am. Soc. Nephrol. 20, 1179–1187 (2009). 19470685 10.1681/ASN.2008050549

[b2] Baker A. J. *et al.* Mesangial cell apoptosis: the major mechanism for resolution of glomerular hypercellularity in experimental mesangial proliferative nephritis. J Clin. Invest 94, 2105–2116 (1994). 7962557 10.1172/JCI117565 PMC294654

[b3] Mauer S. M. *et al.* Structural-functional relationships in diabetic nephropathy. J. Clin. Invest 74, 1143–1155 (1984). 6480821 10.1172/JCI111523 PMC425280

[b4] Steffes M. W., Osterby R., Chavers B. & Mauer S. M. Mesangial expansion as a central mechanism for loss of kidney function in diabetic patients. Diabetes 38, 1077–1081 (1989). 2670639 10.2337/diab.38.9.1077

[b5] Habib R. Nephrotic syndrome in the 1st year of life. Pediatr. Nephrol. 7, 347–353 (1993). 8398639 10.1007/BF00857534

[b6] Saleh H., Schlatter E., Lang D., Pauels H. G. & Heidenreich S. Regulation of mesangial cell apoptosis and proliferation by intracellular Ca^2+^ signals. Kidney Int. 58, 1876–1884 (2000). 11044207 10.1111/j.1523-1755.2000.00359.x

[b7] Stockand J. D. & Sansom S. C. Glomerular mesangial cells: electrophysiology and regulation of contraction. Physiol Rev. 78, 723–744 (1998). 9674692 10.1152/physrev.1998.78.3.723

[b8] Ma R., Pluznick J. L. & Sansom S. C. Ion channels in mesangial cells: function, malfunction, or fiction. Physiology. (Bethesda.) 20, 102–111 (2005). 15772299 10.1152/physiol.00050.2004

[b9] Dryer S. E. & Reiser J. TRPC6 channels and their binding partners in podocytes: role in glomerular filtration and pathophysiology. Am. J Physiol Renal Physiol 299, F689–F701 (2010). 20685822 10.1152/ajprenal.00298.2010 PMC2957253

[b10] Schlondorff J. S. & Pollak M. R. TRPC6 in glomerular health and disease: what we know and what we believe. Semin. Cell Dev. Biol. 17, 667–674 (2006). 17116414 10.1016/j.semcdb.2006.11.003 PMC2705932

[b11] Reiser J. *et al.* TRPC6 is a glomerular slit diaphragm-associated channel required for normal renal function. Nat. Genet. 37, 739–744 (2005). 15924139 10.1038/ng1592 PMC1360984

[b12] Winn M. P. *et al.* A mutation in the TRPC6 cation channel causes familial focal segmental glomerulosclerosis. Science 308, 1801–1804 (2005). 15879175 10.1126/science.1106215

[b13] Ilatovskaya D. V. *et al.* Podocyte injury in diabetic nephropathy: implications of angiotensin II-dependent activation of TRPC channels. Sci. Rep 5, 17637 (2015). 26656101 10.1038/srep17637 PMC4674698

[b14] Graham S. *et al.* Downregulation of TRPC6 protein expression by high glucose, a possible mechanism for the impaired Ca^2+^ signaling in glomerular mesangial cells in diabetes. Am. J. Physiol Renal Physiol 293, F1381–F1390 (2007). 17699555 10.1152/ajprenal.00185.2007

[b15] Qiu G. & Ji Z. AngII-induced glomerular mesangial cell proliferation inhibited by losartan via changes in intracellular calcium ion concentration. Clin. Exp. Med 14, 169–176 (2014). 23459786 10.1007/s10238-013-0232-y PMC4000622

[b16] Kong F. *et al.* Alpha1-Adrenergic Receptor Activation Stimulates Calcium Entry and Proliferation via TRPC6 Channels in Cultured Human Mesangial Cells. Cell Physiol Biochem. 36, 1928–1938 (2015). 26202353 10.1159/000430161

[b17] Liao C. *et al.* The upregulation of TRPC6 contributes to Ca^2+^ signaling and actin assembly in human mesangial cells after chronic hypoxia. Biochem. Biophys. Res. Commun. 421, 750–756 (2012). 22554521 10.1016/j.bbrc.2012.04.075

[b18] Rao A., Luo C. & Hogan P. G. Transcription factors of the NFAT family: regulation and function. Annu. Rev. Immunol. 15, 707–747 (1997). 9143705 10.1146/annurev.immunol.15.1.707

[b19] Horsley V. & Pavlath G. K. NFAT: ubiquitous regulator of cell differentiation and adaptation. J Cell Biol. 156, 771–774 (2002). 11877454 10.1083/jcb.200111073 PMC2173310

[b20] Im S. H. & Rao A. Activation and deactivation of gene expression by Ca^2+^/calcineurin-NFAT-mediated signaling. Mol. Cells 18, 1–9 (2004). 15359117

[b21] Nijenhuis T. *et al.* Angiotensin II contributes to podocyte injury by increasing TRPC6 expression via an NFAT-mediated positive feedback signaling pathway. Am. J Pathol. 179, 1719–1732 (2011). 21839714 10.1016/j.ajpath.2011.06.033 PMC3181349

[b22] Kuwahara K. *et al.* TRPC6 fulfills a calcineurin signaling circuit during pathologic cardiac remodeling. J Clin. Invest 116, 3114–3126 (2006). 17099778 10.1172/JCI27702 PMC1635163

[b23] Schlondorff J., Del C. D., Carrasquillo R., Lacey V. & Pollak M. R. TRPC6 mutations associated with focal segmental glomerulosclerosis cause constitutive activation of NFAT-dependent transcription. Am. J Physiol Cell Physiol 296, C558–C569 (2009). 19129465 10.1152/ajpcell.00077.2008 PMC2660257

[b24] Wang Y. *et al.* Activation of NFAT signaling in podocytes causes glomerulosclerosis. J Am. Soc. Nephrol. 21, 1657–1666 (2010). 20651158 10.1681/ASN.2009121253 PMC3013542

[b25] Ding Y. *et al.* Reactive oxygen species-mediated TRPC6 protein activation in vascular myocytes, a mechanism for vasoconstrictor-regulated vascular tone. J Biol. Chem. 286, 31799–31809 (2011). 21768109 10.1074/jbc.M111.248344 PMC3173128

[b26] Leuner K. *et al.* Hyperforin–a key constituent of St. John’s wort specifically activates TRPC6 channels. FASEB J. 21, 4101–4111 (2007). 17666455 10.1096/fj.07-8110com

[b27] Muller M. *et al.* Specific TRPC6 channel activation, a novel approach to stimulate keratinocyte differentiation. J Biol. Chem. 283, 33942–33954 (2008). 18818211 10.1074/jbc.M801844200 PMC2662218

[b28] Ikeda K. *et al.* Roles of transient receptor potential canonical (TRPC) channels and reverse-mode Na^+^/Ca^2+^ exchanger on cell proliferation in human cardiac fibroblasts: effects of transforming growth factor beta1. Cell Calcium 54, 213–225 (2013). 23827314 10.1016/j.ceca.2013.06.005

[b29] Leuner K. *et al.* Hyperforin modulates dendritic spine morphology in hippocampal pyramidal neurons by activating Ca^2+^-permeable TRPC6 channels. Hippocampus 23, 40–52 (2013). 22815087 10.1002/hipo.22052 PMC3538039

[b30] Leuner K. *et al.* Simple 2,4-diacylphloroglucinols as classic transient receptor potential-6 activators–identification of a novel pharmacophore. Mol. Pharmacol. 77, 368–377 (2010). 20008516 10.1124/mol.109.057513

[b31] Whiteside C., Munk S., Zhou X., Miralem T. & Templeton D. M. Chelation of intracellular calcium prevents mesangial cell proliferative responsiveness. J Am. Soc. Nephrol. 9, 14–25 (1998). 9440082 10.1681/ASN.V9114

[b32] Nagata S. Fas ligand-induced apoptosis. Annu. Rev. Genet. 33, 29–55 (1999). 10690403 10.1146/annurev.genet.33.1.29

[b33] Boyle J. J. Human macrophages kill human mesangial cells by Fas-L-induced apoptosis when triggered by antibody via CD16. Clin. Exp. Immunol. 137, 529–537 (2004). 15320902 10.1111/j.1365-2249.2004.02565.x PMC1809132

[b34] Pinkoski M. J. & Green D. R. Fas ligand, death gene. Cell Death. Differ. 6, 1174–1181 (1999). 10637433 10.1038/sj.cdd.4400611

[b35] Kavurma M. M. & Khachigian L. M. Signaling and transcriptional control of Fas ligand gene expression. Cell Death. Differ. 10, 36–44 (2003). 12655294 10.1038/sj.cdd.4401179

[b36] Merhi F. *et al.* Hyperforin inhibits Akt1 kinase activity and promotes caspase-mediated apoptosis involving Bad and Noxa activation in human myeloid tumor cells. PLoS. One. 6, e25963 (2011). 21998731 10.1371/journal.pone.0025963 PMC3188562

[b37] Hostanska K., Reichling J., Bommer S., Weber M. & Saller R. Hyperforin a constituent of St John’s wort (Hypericum perforatum L.) extract induces apoptosis by triggering activation of caspases and with hypericin synergistically exerts cytotoxicity towards human malignant cell lines. Eur. J Pharm. Biopharm. 56, 121–132 (2003). 12837490 10.1016/s0939-6411(03)00046-8

[b38] Graham S. *et al.* Abundance of TRPC6 protein in glomerular mesangial cells is decreased by ROS and PKC in diabetes. Am. J. Physiol Cell Physiol 301, C304–C315 (2011). 21525431 10.1152/ajpcell.00014.2011 PMC3154551

[b39] Clapham D. E. TRP channels as cellular sensors. Nature 426, 517–524 (2003). 14654832 10.1038/nature02196

[b40] Vazquez G., Wedel B. J., Aziz O., Trebak M. & Putney J. W. Jr. The mammalian TRPC cation channels. Biochim. Biophys. Acta 1742, 21–36 (2004). 15590053 10.1016/j.bbamcr.2004.08.015

[b41] Ohba T. *et al.* Upregulation of TRPC1 in the development of cardiac hypertrophy. J Mol. Cell Cardiol. 42, 498–507 (2007). 17174323 10.1016/j.yjmcc.2006.10.020

[b42] Makarewich C. A. *et al.* Transient receptor potential channels contribute to pathological structural and functional remodeling after myocardial infarction. Circ. Res. 115, 567–580 (2014). 25047165 10.1161/CIRCRESAHA.115.303831 PMC4149870

[b43] Abkhezr M. *et al.* Pleiotropic signaling evoked by tumor necrosis factor in podocytes. Am. J Physiol Renal Physiol 309, F98–108 (2015). 26017975 10.1152/ajprenal.00146.2015

[b44] Nagata S. & Golstein P. The Fas death factor. Science 267, 1449–1456 (1995). 7533326 10.1126/science.7533326

[b45] Kischkel F. C. *et al.* Death receptor recruitment of endogenous caspase-10 and apoptosis initiation in the absence of caspase-8. J Biol. Chem. 276, 46639–46646 (2001). 11583996 10.1074/jbc.M105102200

[b46] Boldin M. P. *et al.* A novel protein that interacts with the death domain of Fas/APO1 contains a sequence motif related to the death domain. J Biol. Chem. 270, 7795–7798 (1995). 7536190 10.1074/jbc.270.14.7795

[b47] Chinnaiyan A. M., O’Rourke K., Tewari M. & Dixit V. M. FADD, a novel death domain-containing protein, interacts with the death domain of Fas and initiates apoptosis. Cell 81, 505–512 (1995). 7538907 10.1016/0092-8674(95)90071-3

[b48] Kischkel F. C. *et al.* Cytotoxicity-dependent APO-1 (Fas/CD95)-associated proteins form a death-inducing signaling complex (DISC) with the receptor. EMBO J 14, 5579–5588 (1995). 8521815 10.1002/j.1460-2075.1995.tb00245.x PMC394672

[b49] Kalivendi S. V. *et al.* Doxorubicin activates nuclear factor of activated T-lymphocytes and Fas ligand transcription: role of mitochondrial reactive oxygen species and calcium. Biochem. J 389, 527–539 (2005). 15799720 10.1042/BJ20050285 PMC1175131

[b50] Chai W. R., Wang Q. & Gao H. B. NFAT2 is implicated in corticosterone-induced rat Leydig cell apoptosis. Asian J Androl 9, 623–633 (2007). 17712479 10.1111/j.1745-7262.2007.00257.x

[b51] Srivastava R. K., Sasaki C. Y., Hardwick J. M. & Longo D. L. Bcl-2-mediated drug resistance: inhibition of apoptosis by blocking nuclear factor of activated T lymphocytes (NFAT)-induced Fas ligand transcription. J Exp. Med 190, 253–265 (1999). 10432288 10.1084/jem.190.2.253 PMC2195578

[b52] Jayanthi S. *et al.* Calcineurin/NFAT-induced up-regulation of the Fas ligand/Fas death pathway is involved in methamphetamine-induced neuronal apoptosis. Proc. Natl. Acad. Sci. USA 102, 868–873 (2005). 15644446 10.1073/pnas.0404990102 PMC545515

[b53] Tu P., Gibon J. & Bouron A. The TRPC6 channel activator hyperforin induces the release of zinc and calcium from mitochondria. J Neurochem. 112, 204–213 (2010). 19845832 10.1111/j.1471-4159.2009.06446.x

[b54] Friedland K. & Harteneck C. Hyperforin: To Be or Not to Be an Activator of TRPC(6). Rev. Physiol Biochem. Pharmacol. 169, 1–24 (2015). 26384487 10.1007/112_2015_25

[b55] Sell T. S., Belkacemi T., Flockerzi V. & Beck A. Protonophore properties of hyperforin are essential for its pharmacological activity. Sci. Rep 4, 7500 (2014). 25511254 10.1038/srep07500 PMC4266863

[b56] Babcock D. F., Herrington J., Goodwin P. C., Park Y. B. & Hille B. Mitochondrial participation in the intracellular Ca^2+^ network. J Cell Biol. 136, 833–844 (1997). 9049249 10.1083/jcb.136.4.833 PMC2132502

[b57] Walsh C. *et al.* Modulation of calcium signalling by mitochondria. Biochim. Biophys. Acta 1787, 1374–1382 (2009). 19344663 10.1016/j.bbabio.2009.01.007

[b58] Adebiyi A., Soni H., John T. A. & Yang F. Lipid rafts are required for signal transduction by angiotensin II receptor type 1 in neonatal glomerular mesangial cells. Exp. Cell Res. 324, 92–104 (2014). 24662198 10.1016/j.yexcr.2014.03.011

[b59] Adebiyi A. RGS2 regulates urotensin II-induced intracellular Ca^2+^ elevation and contraction in glomerular mesangial cells. J. Cell Physiol 229, 502–511 (2014). 24105430 10.1002/jcp.24470 PMC11250777

